# Diagnostic and predictive values of pyroptosis-related genes in sepsis

**DOI:** 10.3389/fimmu.2023.1105399

**Published:** 2023-02-02

**Authors:** Xuesong Wang, Zhe Guo, Ziyi Wang, Haiyan Liao, Ziwen Wang, Feng Chen, Zhong Wang

**Affiliations:** ^1^ School of Clinical Medicine, Tsinghua University, Beijing, China; ^2^ Department of General Medicine, Beijing Tsinghua Changgung Hospital affiliated to Tsinghua University, Beijing, China

**Keywords:** sepsis, pyroptosis, machine learning (ML), immune landscape, MALT1 gene

## Abstract

**Background:**

Sepsis is an organ dysfunction syndrome caused by the body’s dysregulated response to infection. Yet, due to the heterogeneity of this disease process, the diagnosis and definition of sepsis is a critical issue in clinical work. Existing methods for early diagnosis of sepsis have low specificity.

**Aims:**

This study evaluated the diagnostic and predictive values of pyroptosis-related genes in normal and sepsis patients and their role in the immune microenvironment using multiple bioinformatics analyses and machine-learning methods.

**Methods:**

Pediatric sepsis microarray datasets were screened from the GEO database and the differentially expressed genes (DEGs) associated with pyroptosis were analyzed. DEGs were then subjected to multiple bioinformatics analyses. The differential immune landscape between sepsis and healthy controls was explored by screening diagnostic genes using various machine-learning models. Also, the diagnostic value of these diagnosis-related genes in sepsis (miRNAs that have regulatory relationships with genes and related drugs that have regulatory relationships) were analyzed in the internal test set and external test.

**Results:**

Eight genes (CLEC5A, MALT1, NAIP, NLRC4, SERPINB1, SIRT1, STAT3, and TLR2) related to sepsis diagnosis were screened by multiple machine learning algorithms. The CIBERSORT algorithm confirmed that these genes were significantly correlated with the infiltration abundance of some immune cells and immune checkpoint sites (all *P*<0.05). SIRT1, STAT3, and TLR2 were identified by the DGIdb database as potentially regulated by multiple drugs. Finally, 7 genes were verified to have significantly different expressions between the sepsis group and the control group (*P*<0.05).

**Conclusion:**

The pyroptosis-related genes identified and verified in this study may provide a useful reference for the prediction and assessment of sepsis.

## 1 Introduction

Sepsis is the systemic response to severe infection. It is the main cause of death in critically ill patients, and its incidence keeps rising (1). Sepsis can be caused by an infection that originates at multiple sites and most patients with sepsis are infected by pathogenic microorganisms. Patients with severe underlying diseases (such as diabetes, leukemia, etc.) are more likely to suffer from this condition. Sepsis is the leading cause of death in hospitalized patients (2), and previous studies have shown that 30% of inpatient mortality is caused by sepsis (3). Yet, due to the heterogeneity of the development of this disease, the diagnosis and definition of sepsis are critical issues in clinical work. In early 2016, the new definitions of sepsis changed dramatically; briefly, greater emphasis was placed on the diagnostic value of “organ dysfunction”, described as an acute increase in total Sequential Organ Failure Assessment (SOFA) score. According to the new sepsis 3.0 diagnostic criteria, sepsis is defined as “life-threatening organ dysfunction caused by the imbalance of the body’s response to infection” (4). Therefore, the diagnosis of sepsis is delayed compared with previous systemic inflammatory response syndrome (SIRS) scores. However, screening for patients at risk of developing sepsis may lead to more effective interventions that can greatly reduce the incidence of sepsis, mortality, and medical costs of infected patients (5). Therefore, early diagnosis and prevention of sepsis may be one of the main means to prevent and control sepsis in the future.

Pyroptosis is a new type of programmed cell death with lytic and pro-inflammatory characteristics. It is mediated by the cysteine aspartate-specific protein kinases 1, 4, 5, and 11, and its final step is dependent on the activity of the gasdermin family proteins that form pores in the cell membrane (6, 7). Pyroptosis is characterized by cell swelling, changes in plasma membrane permeability, and inflammatory release (8). Inflammasomes have a crucial role in the occurrence and development of sepsis (9). Studies have shown that inflammation-damaged and infected cells in sepsis patients can release pathogens through pyroptosis, which is then promptly eliminated by various immune cells (10). Therefore, pyroptosis can help to fight sepsis-induced infection to a certain extent. Some pyroptosis-related genes have been confirmed to be closely related to sepsis. For example, *GSDMD* has a vital role in sepsis by initiating pyroptosis and activating immune response (11, 12). However, the role of many pyroptosis-related genes in sepsis remains unclear.

In this study, we evaluated the diagnostic and predictive values of pyroptosis-related genes in normal and sepsis tissue and their role in the immune microenvironment using machine-learning methods.

## 2 Methods

### 2.1 Differential expression analysis of genes

The Limma package of R language was used to analyze the differential expression of whole genes (13). The filtering conditions were set as a *P*-value< 0.05 and an absolute value of logFC > 0.2.

### 2.2 Construction of protein-protein interaction network

A PPI network was constructed to explore the interaction among DEGs and the intersection genes of pyroptosis-related genes. Specifically, the PPI network was obtained using protein interaction relationships in the STRING database (14) and the highest confidence interaction score was set to 0.4. Then, the PPI visualization results were obtained using Cytoscape software.

### 2.3 Gene ontology and functional enrichment analysis

Gene Ontology (GO) enrichment analysis (http://www.geneontology.org) (15) and Kyoto Encyclopedia of Genes and Genomes (KEGG) enrichment analysis (www.genome.jp/kegg/) (16) were used to determine the gene biological functions. The GO term is composed of three parts, i.e., biological process (BP), cellular component (CC), and molecular function (MF). Ultimately, significant pathways with *P*<0.05 were selected.

### 2.4 Construction of diagnostic model construction

The machine learning methods used for diagnostic model construction and the relevant package in Scikit-learn (17) were used. These packages’ specific parameter setting ranges are presented in the algorithms.

#### 2.4.1 Random Forest algorithm

The RF algorithm was applied to calculate the importance of genes (18). For each decision tree in the RF, the corresponding out-of-pocket (OOB) data was used to calculate its out-of-pocket data error. The error value could be denoted as *errorOOB*1 . Next, by randomly adding noise to the out-of-pocket data *X* , the out-of-pocket data error errorOOB2 could be calculated again. Finally, the calculation formula of the importance of out-of-pocket data *X* (*feature*(*X*) ) was obtained:


(1) $$feature\left(X\right)=\sum^{}(err00B2-errOOB1) /Ntree$$

where Ntree represents the number of decision trees. When ranking feature importance, the specific parameters of RF were set as follows: the number of decision trees was selected between 100 and 1000, and the default values were used for other parameters.

#### 2.4.2 Adaboost algorithm

The Adaboost algorithm was used to construct the diagnostic model. The AdaBoost algorithm is a boosting algorithm (19). In model training, each sample was given a probability (weight) that indicates that it was selected into the training set in a specific classifier. If the sample was accurately classified, the probability of it being chosen for the next training set was reduced. Conversely, the probability of being selected for the next training set was considerably improved.

In a binary classification training set (*X*={(*x*_1_,*y*_1_),(*x*_2_,*y*_2_),⋯,(*x*_*n*_,*y*_*n*_)} }, *x*_*i*_ and *y*_*i*_ represent the sample points and their corresponding binary classification results *y*_*i*_ . The algorithm first initializes the weights of the training set, and the weight redistribution value of the MTH weak learner is *D*_*m*_=(*w*_*m*1_,⋯,*w*_*mi*_,⋯,*w*_*mN*_) . Here, $w_{mi}=\frac{1}{N},i=1,2,\cdots ,N$ . Next, the weight redistribution values of the training set are updated for the *M* learners. Let *G*_*m*_(*x*) represent the base classifier of the training set with weight distribution *D*_*m*_ . Then the classification error rate *e*_*m*_ of *G*_*m*_(*x*) on the training set can be calculated as follows:


(2) $$e_{m}=\sum_{i=1}^{N}W_{mi}I(G_{m}(x_{i})¹y_{i})$$

the value of *I*(*G*_*m*_(*x*_*i*_)≠*y*_*i*_) is 0 or 1, representing correct and incorrect classification, respectively. Next, the weight coefficient α_m of the classifier was calculated.


(3) $$\alpha_{m}=\frac{1}{2}\text{log}\frac{1-e_{m}}{e_{m}}$$

Finally, the final classifier *G*(*x*) was obtained by updating the weight redistribution value of the training set.


$$\begin{array}{c} D_{m+1}=\left(w_{m+11},w_{m+12},\cdots ,w_{m+1N}\right)\\ w_{m+1i}=\frac{w_{mi}}{Z_{m}}exp\left(-\alpha_{m}y_{i}G_{m}\left(x_{i}\right)\right),i=1,2,\cdots N \end{array}$$

(4) $$G_{\left(x\right)}=sign({\sum_{m=1}^{M}\alpha }_{m}G_{m}(x))$$

The specific parameters of Adaboost were set as follows: the number of decision trees was selected between 100 and 300, the learning rate was determined from two values of 0.01 and 0.1, and the default values were used for other parameters.

#### 2.4.3 Logistic Regression algorithm

LR is a probabilistic nonlinear regression model that can be used for binary classification tasks. Its prediction function is shown below.


(5) $$\text{y}=\frac{1}{1+e^{-\left(w^{T}x+b\right)}}$$

where *w* and *b* are the weight and bias vectors, respectively. In this paper, the specific parameters of LR were set as follows: the reciprocal of the regularization strength was selected between 0.1 and 3.1, and the default values were used for other parameters.

#### 2.4.4 Deep Neural Network algorithm

DNN is an extension based on perceptron. It can be understood as a neural network with many hidden layers (20). The layers of the DNN are fully connected, and any neuron in layer *i* must be connected to any neuron in layer *i*+1 . The forward propagation algorithm of DNN uses several weight matrices *W* and bias vectors *b* to perform a series of nonlinear operations with the input value vector *x* where the output value *a* ^*l*^ of layer *l* can be expressed as the following equation.


(6) $$a^{l}=\sigma \left(W^{l}a^{l-1}+b^{l}\right)\left(l=2,\ldots ,L\right)$$

where *W* ^*l*^ and *b* ^*l*^ represent the *l*−*th* layer’s weight matrix and bias vector, respectively; *L* represents the total number of layers, and 6A represents the activation function. Next, the DNN algorithm updates *W* and *b* by backpropagation. The loss function *J*(*W*,*b*,*x*,*y*) in the backpropagation process can be expressed as the following equation.


(7) $$J\left(W,b,x,y\right)=\frac{1}{2}{\left\|a^{L}-y\right\|}_{2}^{2}$$

where *a* ^*L*^ and *y* represent the output of the output layer and the sample, respectively. The *W* and *b* of each layer can be obtained by gradient descent. In this paper, the specific parameters of DNN were set as follows: the number of hidden layers was 2, the number of neurons in the two hidden layers was 8 or 16, the regularization parameters were selected in 0.001 and 0.01, and the learning rate was selected in ‘constant’ and ‘adaptive.’ Default values were used for other parameters.

#### 2.4.5 Performance evaluation of diagnostic model

The evaluation method of the diagnostic model included calculating the model’s sensitivity, specificity, and f1 score, using the following formulas:


(8) $$Sensitivity_{i}=\frac{TP_{i}}{TP_{i}+FN_{i}}$$

(9) $$Specificity_{i}=\frac{TN_{i}}{TN_{i}+FP_{i}}$$

(10) $$F1{\text{score}}_{i}=\frac{2\times TP_{i}}{2\times TP_{i}+FP_{i}+FN_{i}}$$

where *TP*_*i*_ and *TN*_*i*_ are the true positive and true negative of class *i* , respectively, and *FP*_*i*_ and *FN*_*i*_ are the false positive and false negative of class *i* , respectively.

### 2.5 Immune infiltration analysis

To explore the relationship between diagnosis-related genes and immune cell-related expression, the CIBERSORT algorithm was used to calculate the proportion of different immune cell types in significant samples of GSE26440 dataset (*P*<0.05) (21). Furthermore, the infiltration abundance of 22 kinds of immune cells was obtained, and the correlation between the diagnosis-related genes and the content of 22 immune cells was calculated using the Spearman correlation coefficient. In addition, based on the dataset GSE26440, we used Pearson to calculate the correlation between 47 immune test sites and diagnosis related genes. Finally, we used the external dataset GSE12904 to verify the expression of diagnostic genes.

### 2.6 Construct diagnostic gene-miRNA network

To explore the regulatory relationship between diagnostic genes and miRNAs, miRTarBase (http://mirtarbase.cuhk.edu.cn/php/index.php), miRDB (http://mirdb.org/), and TargetScan (http://www.targetscan.org/vert_72/) databases were queried for miRNAs that have a regulatory relationship with diagnostic genes, and the intersection genes of the three databases were selected to construct a diagnostic gene-miRNA network.

### 2.7 Identifying potential drugs

To identify potential therapeutic drugs for sepsis, Drug-Gene Interaction Database (DGIdb) version 3.0.2 (https://www.dgidb.org) (22) was used to analyze the interaction between the diagnostic genes and related drugs. In addition, we searched the DGIdb to predict potential drugs or molecular compounds that interact with DEG.

### 2.8 qRT-PCR

The whole blood samples of six patients with sepsis and six healthy people were collected from Beijing Tsinghua Changgung Hospital (Beijing, China). Demographic information could be found in **Table S1** of Supplementary materials. The screening criteria for sepsis patients were based on sepsis 3.0 (4). Whole blood samples were obtained from patients, and the quantitative real-time polymerase chain reaction (RT-PCR) was performed (Primer list could be found in **Table S2** of Supplementary materials). None of the patients had a history of autoimmune disorders, neoplastic diseases, or oral immunosuppressants. This study was approved by the Ethics Committee (NCT05095324).

## 3 Results

### 3.1 Microarray data set acquisition and data processing

Microarray data sets related to sepsis were downloaded from the GEO database. GSE26440 was selected as the internal training and test set and GSE13904 as the external test set. GSE26440 contains 130 whole-blood RNA samples from 98 samples with septic shock and 32 healthy controls. GSE13904 includes 158 samples with sepsis and 18 healthy controls.

**Figure 1:**
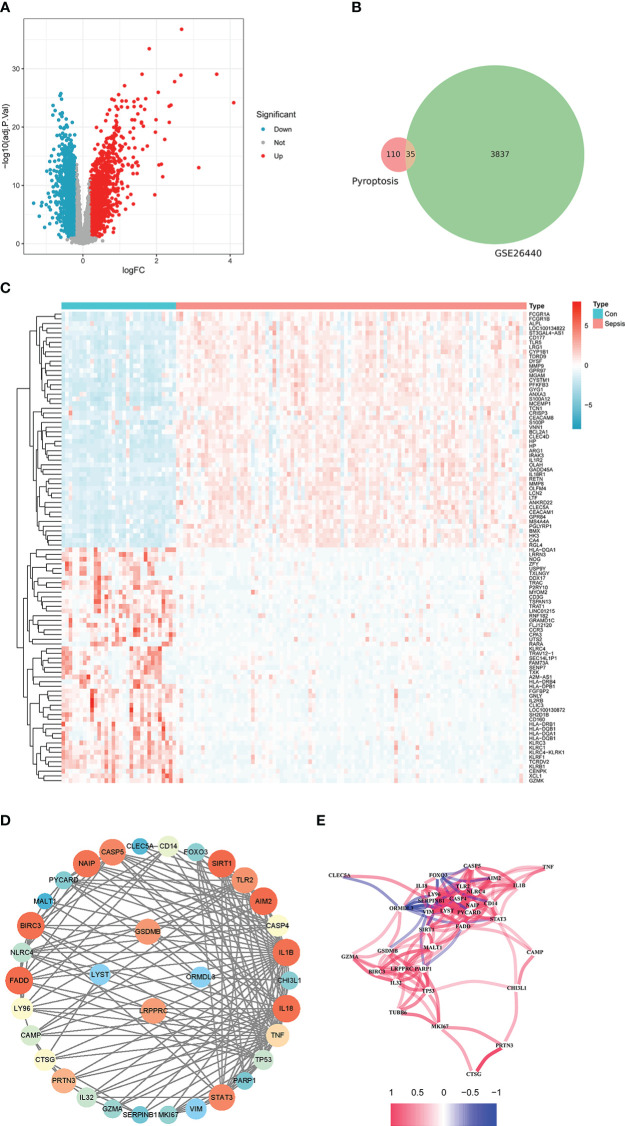
Differential analysis of the internal training set. **(A)** is the volcano plot from the difference analysis. **(B)** is a Venn diagram of pyroptosis-related genes plotted against the differentially expressed genes of the training set. **(C)** is the expression heatmap of the top 50 up-regulated and down-regulated genes by absolute logFC. **(D)** is the PPI network constructed by the intersection genes of DEGs and pyroptosis-related genes. **(E)** is a correlation network plot among intersecting genes.

GPL570-55999 platform file was used to convert gene symbols for the two GEO datasets. When multiple probes were mapped to the same gene symbol, the average value of the probes was selected as the gene expression value. Furthermore, 3837 DEGs were selected for differential expression analysis on the GSE26440 dataset. Of these, 2302 were down-regulated (**Figure 1**, blue dots) and 1535 were up-regulated (**Figure 1**, red dots). Next, a heat map of the top 50 up-regulated genes and the top 50 down-regulated genes among the differentially expressed genes were drawn (**Figure 1**).

Also, 110 genes related to pyroptosis were collected from previous studies. There were 35 intersection genes of DEGs and pyroptosis-related genes, which could be visualized by the Venn diagram in this paper (**Figure 1**). The PPI network of intersecting genes is shown in **Figure 1**. Among them, the node color’s depth and area represent the number of connections with the gene. **Figure 1** shows the Pearson correlation coefficient (PCC) network plot among intersecting genes. Most of the 35 intersecting genes have strong correlations.

**Figure 2:**
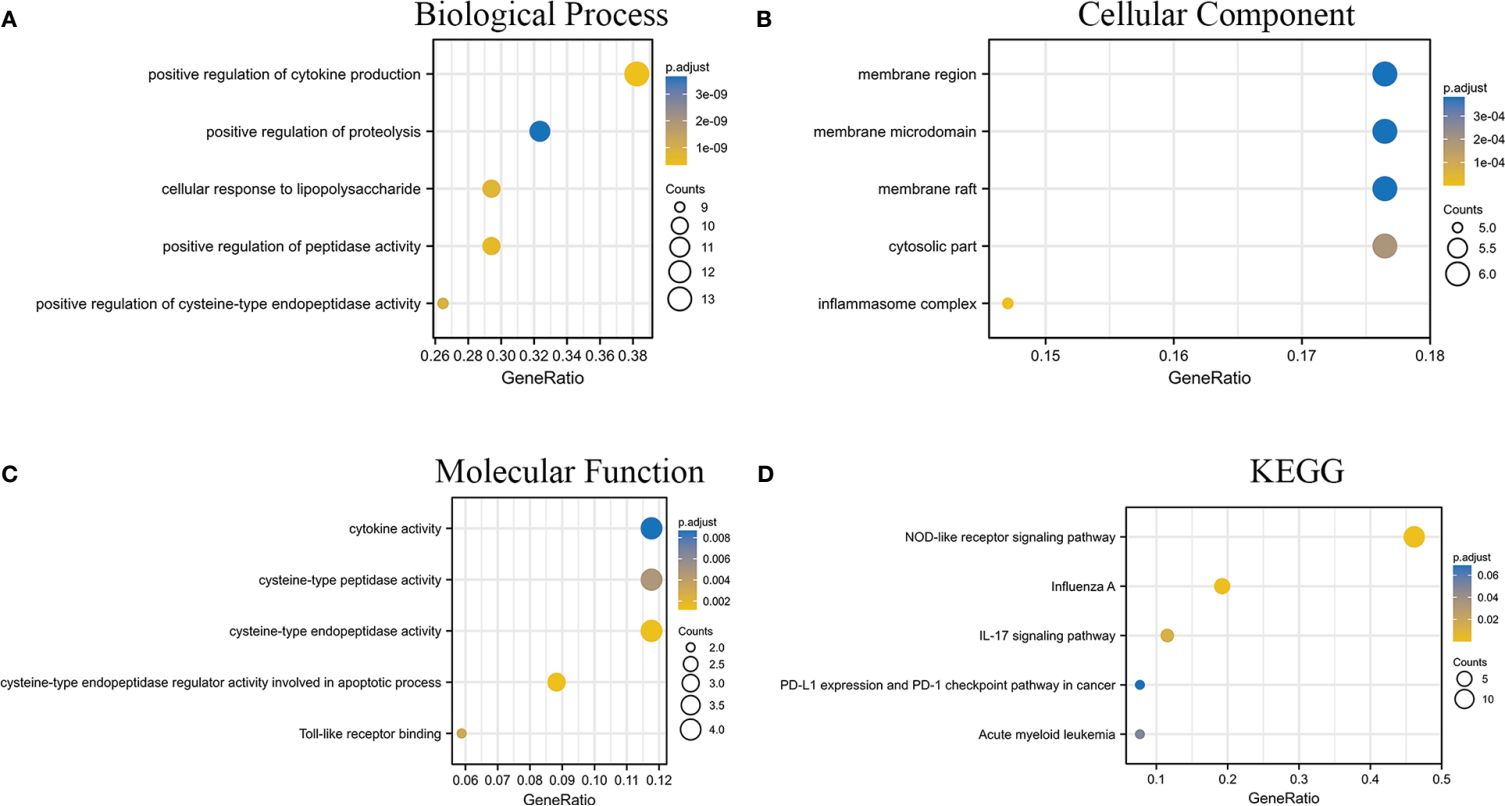
GO and KEGG enrichment analysis results of intersecting genes. **(A–C)** are the analysis results of BP, CC, and MF in GO, respectively. **(D)** is the result of the KEGG enrichment analysis.

In addition, to explore the pathway information involved in the intersection genes, we drew a bubble plot of significant pathways with the BP (**Figure 2**), CC (**Figure 2**), and MF (**Figure 2**) entries in the GO enrichment analysis and the KEGG enrichment analysis (**Figure 2**), respectively.

### 3.2 Gene screening and diagnostic model construction

**Figure 3:**
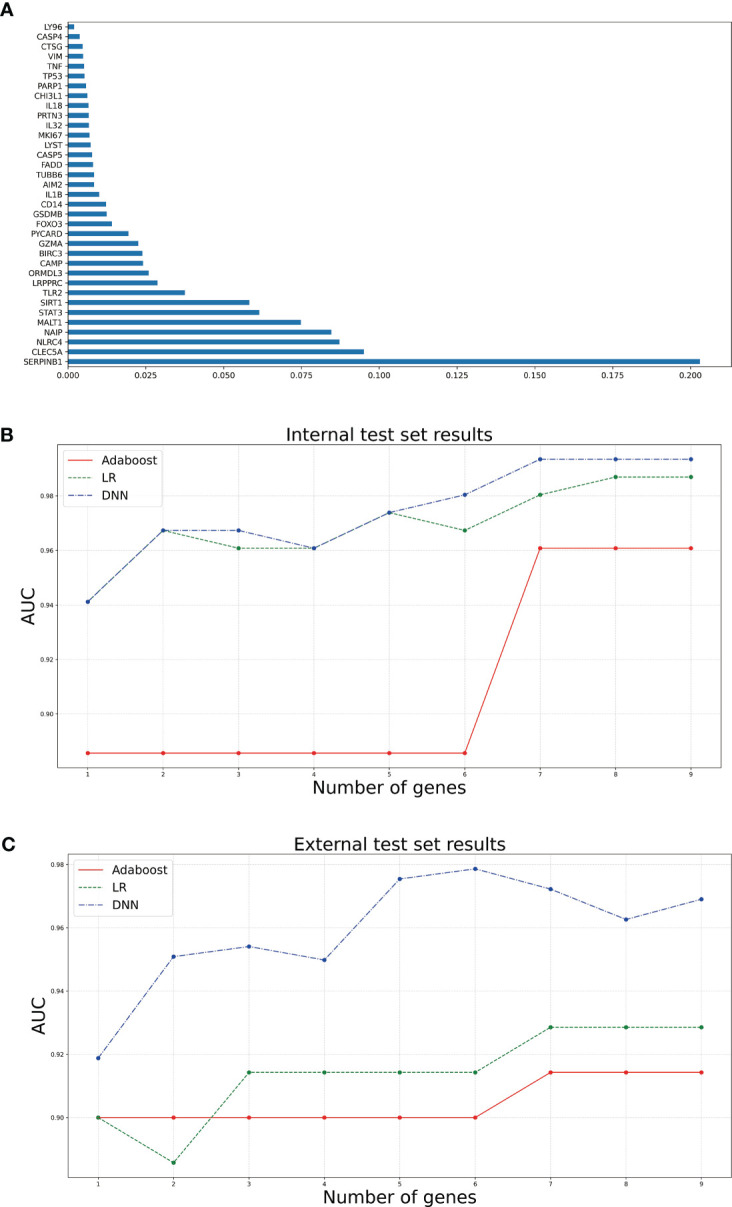
Bar and line graphs associated with diagnostic model construction. **(A)** is a bar graph of the 35 genes ranked by feature importance using RF algorithm. **(B, C)** are line plots of the AUCs corresponding to different numbers of top genes selected using the three classifiers on the internal and external test sets, respectively.

To screen out the genes related to diagnosis from the 35 intersection genes, RF was used to rank the importance of the 35 features. The mean weight of 35 genes was 0.0285. We retained nine genes larger than the weight mean (**Figure 3A**). Next, we constructed sepsis diagnostic models using Adaboost, LR, and DNN classifiers to identify the pyroptosis-related genes related to diagnosis. Specifically, the top 1-top 9 genes on the training set were input to the three classifiers. After setting the corresponding classifier parameter range, the optimal parameters were selected using ten-fold cross-validation, and then the AUC results on the internal test set were obtained (**Figure 3B**). The AUC of the DNN classifier reached 0.9935 when using the top 9 genes. To further confirm the diagnostic power of the signature genes, the results were validated using the GSE13904 dataset (**Figure 3C**). The DNN classifier can reach an AUC of 0.9786 when using the top 8 genes. **Table 1** presents the model performance metrics when the AUC is maximized using the DNN model.

**Table 1:** AUC, sensitivity, specificity, and f1 score of the diagnostic model consisting of multiple genes on the internal and external test sets.

Test set type	Number of genes	AUC	Sensitivity	Specificity	f1 score
Internal test set	9	0.9935	1	0.8889	0.9714
External test set	6	0.9786	0.9423	1	0.9703

**Figure 4:**
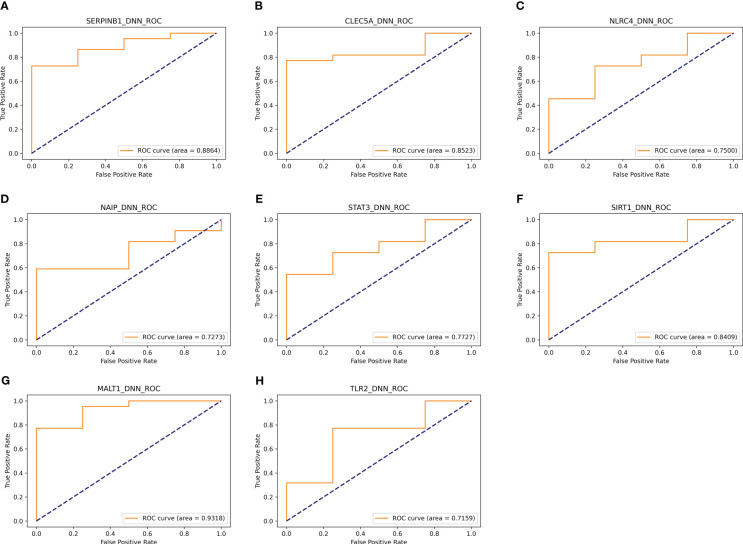
Results of separate validation of the top eight genes on the internal validation set. **(A–H)** are ROC curves for the top 8 genes, respectively.

**Figure 5:**
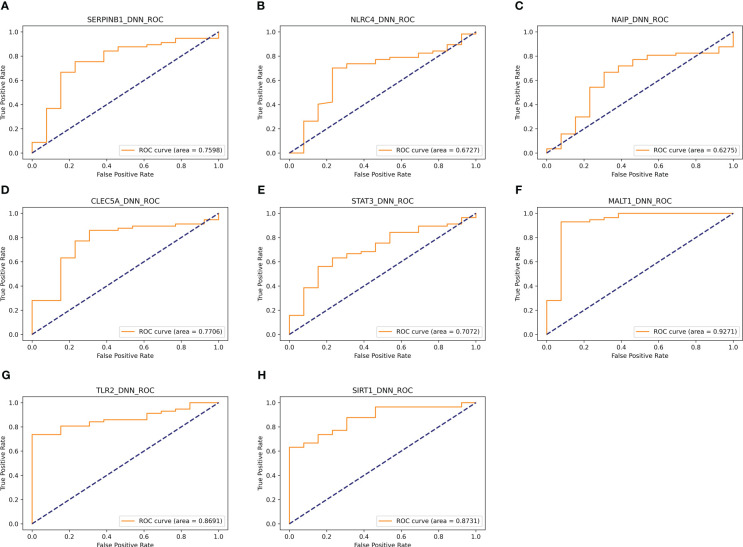
Results of separate validation of the top eight genes on the external validation set. **(A–H)** are ROC curves for the top 8 genes, respectively.

In addition, eight genes were further validated in the internal test set (**Figures 4A–H**) and external test set (**Figures 5A–H**) independently using the DNN classifier. **Table 2** and **Table 3** state the diagnostic model’s performance on the internal and external test sets, respectively. LRPPRC was not considered because it presented false positives in diagnostic model construction.

**Table 2:** AUC, sensitivity, specificity, and f1 score of the diagnostic model composed of a single gene on the internal test set.

Gene symbol	AUC	Sensitivity	Specificity	f1 score
SERPINB1	0.8664	0.9412	0.3333	0.8205
NLRC4	0.75	0.8889	0.25	0.8
NAIP	0.7273	0.8889	0.25	0.8
CLEC5A	0.8523	0.9444	0.375	0.85
STAT3	0.7727	0.8636	0.25	0.8636
MALT1	0.9318	0.9524	0.6	0.9302
TLR2	0.7159	0.85	0.1667	0.8095
SIRT1	0.8409	0.8571	0.2	0.8372

**Table 3:** AUC, sensitivity, specificity, and f1 score of the diagnostic model composed of a single gene on the external test set.

Gene symbol	AUC	Sensitivity	Specificity	f1 score
SERPINB1	0.7598	0.902	0.4211	0.8519
NLRC4	0.6727	0.8333	0.25	0.8108
NAIP	0.6275	0.8364	0.2667	0.8214
CLEC5A	0.7706	0.9245	0.5294	0.8909
STAT3	0.7072	0.8421	0.3077	0.8421
MALT1	0.9271	0.9808	0.6667	0.9358
TLR2	0.8691	0.8621	0.4167	0.8696
SIRT1	0.8731	0.873	0.7143	0.9167

### 3.3 Immune infiltration and the landscape of immune checkpoint genes in sepsis

**Figure 6:**
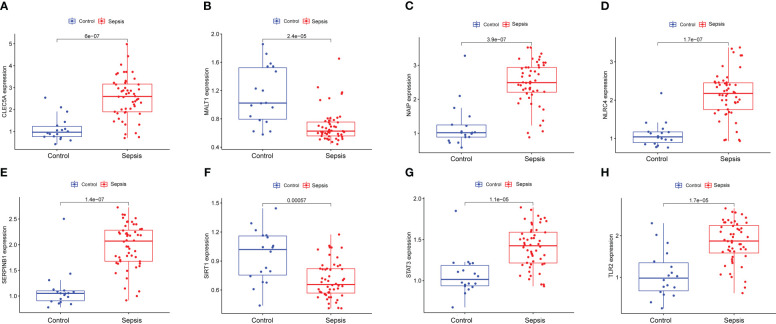
Expression of eight diagnostically relevant genes in sepsis patients and healthy controls of CLEC5A **(A)**, MALT1 **(B)**, NAIP **(C)** NLRC4 **(D)**, SERPINB1 **(E)**, SIRT1 **(F)**, STAT3 **(G)** and TLR2 **(H)** relative to GAPDH. Median ± maximum/minimum.

**Figure 7:**
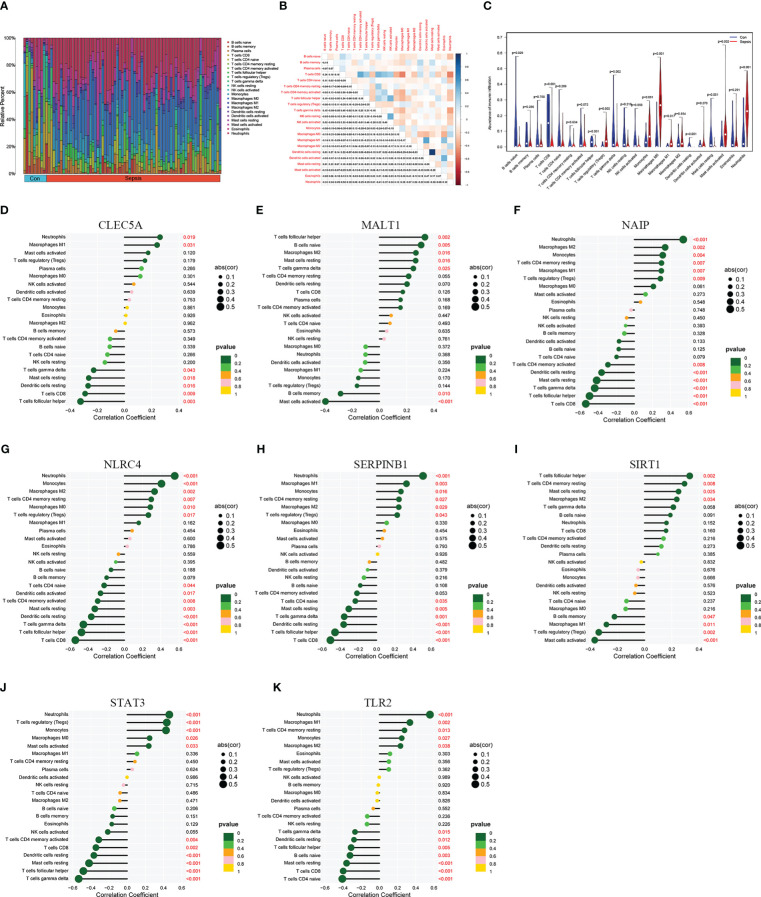
Immune infiltration landscape in sepsis and healthy controls. **(A)** is a bar graph of the proportions of 22 immune cells in sepsis and controls. **(B)** is a heatmap of correlations among 22 immune cells. **(C)** shows the differential expression of different immune cell infiltration abundance in sepsis and control. **(D–K)** are lollipop plots of CLEC5A, MALT1, NAIP, NLRC4, SERPINB1, SIRT1, STAT3, and TLR2 correlations with immune cells, respectively.

To explore the immune infiltration of sepsis, the CIBERSORT algorithm was used to analyze the differences in peripheral blood immune cells between the significant sepsis samples and healthy control samples. The boxplots in **Figures 6A–H** showed the expression of eight diagnostically relevant genes in sepsis patients and healthy controls. Eight genes significantly differed between the two groups. **Figure 7A** shows the proportional expression of 22 immune cells in Control samples and sepsis samples. **Figure 7B** shows a heatmap of correlations between immune cells. Resting mast cells and resting dendritic cells had the strongest positive correlation (r=0.94). Conversely, Macrophages M0 and T cells CD8 had the strongest negative correlation (r=0.5). **Figure 7C** shows the differential expression of different immune cell marker expression types between sepsis and controls. Among them, the expression levels of B cells naive, B cells memory, T cells CD8, activated T CD4 memory cells, T cells follicular helper, regulatory T cells, T cells gamma delta, activated NK cells, macrophages M0, macrophages M1, resting dendritic cells, resting and cells activated mast cells, and neutrophils were significantly different between the two groups (all P<0.05). In addition, we showed the lollipop plots of eight sepsis diagnosis-related genes (CLEC5A, MALT1, NAIP, NLRC4, SERPINB1, SIRT1, STAT3, and TLR2) associated with 22 immune cells in **Figures 7D–K**.

**Figure 8:**
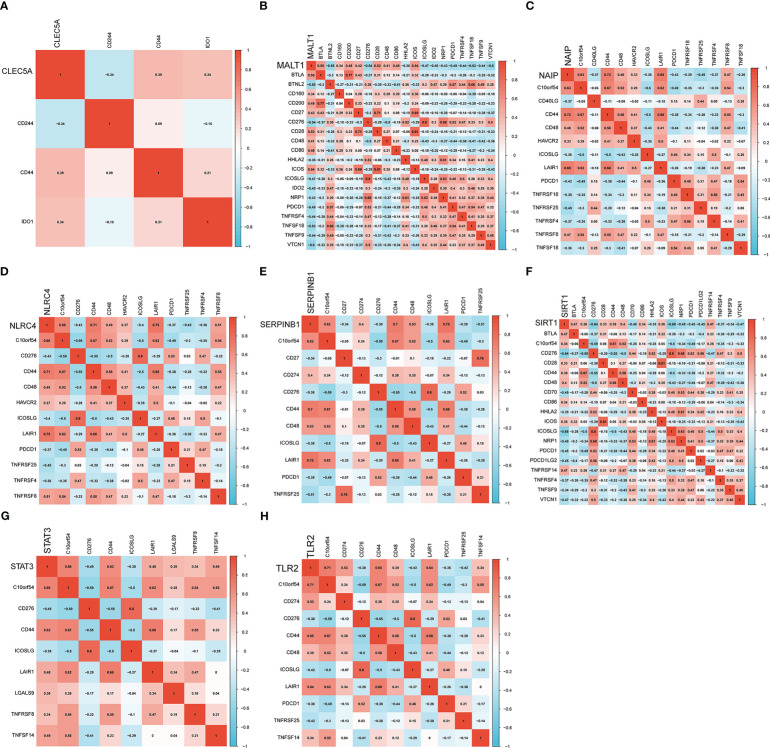
Heatmap of the association of eight genes with immune checkpoints. **(A–H)** are correlation heatmaps with significant associations between diagnosis-related genes (CLEC5A, MALT1, NAIP, NLRC4, SERPINB1, SIRT1, STAT3 and TLR2) and immune checkpoints.

Next, we analyzed the association of diagnostic genes with 47 immune checkpoints. Specifically, the immune checkpoints were retained with significant associations (*P*<0.001) with eight diagnosis-related genes and a heatmap of the associations between them was created (**Figures 8A–H**). For CLEC5A, the immune checkpoints with the strongest positive/negative correlation were CD44 (r=0.39) and CD244 (r=-0.34), respectively. For MALT1, the immune checkpoints with the strongest positive/negative correlation were BTLA (r=0.55) and BTNL2 (r=-0.55), respectively. For NAIP, the immune checkpoints with the strongest positive/negative correlation were CD44 (r=0.72) and TNFRSF25 (r=-0.49), respectively. For NLRC4, the immune checkpoints with the strongest positive/negative correlation were LAIR1 (r=0.75) and TNFRSF25 (r=-0.45), respectively. For SERPINB1, the immune checkpoints with the strongest positive/negative correlation were LAIR1 (r=0.75) and TNFRSF25 (r=-0.51), respectively. For SIRT1, the immune checkpoints with the strongest positive/negative correlation were BTLA (r=0.47) and ICOSLG (r=-0.68), respectively. For STAT3, the immune checkpoints with the strongest positive/negative correlation were C10orf54 (r=0.66) and CD276 (r=-0.49), respectively. For TLR2, the immune checkpoints with the strongest positive/negative correlation were C10orf54 (r=0.71) and ICOSLG/TNFRSF25 (r=-0.49), respectively.

To determine the regulatory relationship among eight diagnosis-related genes and miRNAs, three miRNA databases were used to query the regulatory relationship among diagnosis-related genes. We took the intersection genes of the three database results and mapped the regulatory network of diagnosis-related genes and miRNAs (**Figure S1A**). More miRNAs regulated STAT3, NAIP, and SIRT1. Furthermore, we applied DGIdb to identify potential drugs or molecular compounds that could reverse the expression of diagnostically relevant genes (**Figure S1B**). Various drugs might regulate SIRT1, STAT3, and TLR2. The inhibitory effects of some compounds on sepsis were demonstrated. Rare ginsenosides Rk1 and Rg5 have an inhibitory effect on high mobility group box 1 (HMGB1)-mediated sepsis (23). HMGB1 is a cytokine present in the late stage of sepsis. The up-regulation of SIRT1 by ethyl pyruvate (EP) may promote the deacetylation of HMGB1, thereby reducing the release of HMGB1 from lipopolysaccharide (LPS)-activated macrophages (24). Lee et al. demonstrated the inhibitory effect of ginsenoside Rh1 on HMGB1-mediated sepsis response (25). In addition, celecoxib combined with low-dose antibiotics in treating sepsis in mice can significantly reduce the bacterial load and inflammatory markers in different organs of mice (26).

### 3.4 Expression validation of diagnosis-related genes

**Figure 9:**
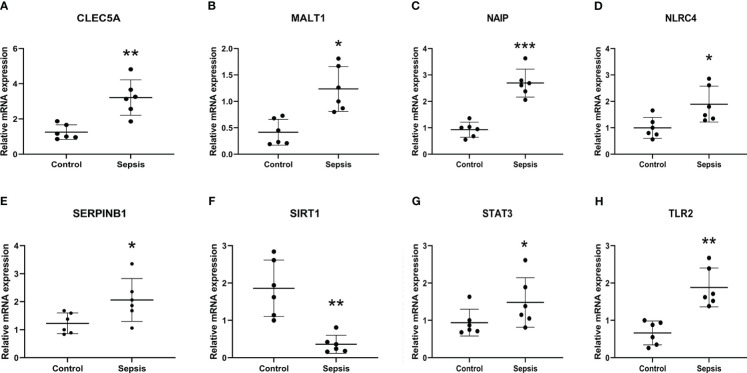
Transcriptional expression in whole blood from sepsis patients and healthy controls of CLEC5A **(A)**, MALT1 **(B)**, NAIP **(C)** NLRC4 **(D)**, SERPINB1 **(E)**, SIRT1 **(F)**, STAT3 **(G)** and TLR2 **(H)** relative to GAPDH. Mean ± standard deviation, ***P<0.001, **P<0.01, *P<0.05 vs. the Control group.

qRT-PCR was used to verify the expression levels of eight diagnosis-related genes between the sepsis group and the control groups, Demographic information could be found in **Table S1** of **Supplementary materials**. The results showed that, except for STAT3, the expression of other genes was significantly different between the two groups (**Figure 9**), which was consistent with the results of bioinformatics analysis. Therefore, further exploration of their roles in sepsis is of great significance for diagnosing and treating sepsis.

## 4 Discussion

Sepsis is a syndrome that often occurs due to various infections. Its incidence is increasing year by year. Therefore, early diagnosis of sepsis is necessary. In addition, the role of pyroptosis-related genes in the occurrence and development of sepsis is still unclear. Therefore, this paper aims to explore the genes related to the diagnosis of sepsis and pyroptosis.

We performed enrichment analyses after the intersection of DEGs and pyroptosis-related genes and obtained multiple immune-related pathways. The GO and KEGG enrichment analysis showed that most of these genes were enriched in immune and tumor progression-related pathways. Danielski et al. systematically reviewed the NLRP3 inflammasome and its role in developing sepsis, and incorrect regulation of NF-κB has been associated with inflammation and autoimmunity (27). Zhou et al. found that NF-κB activation is involved in cardiomyocyte apoptosis, cardiomyocyte autophagy, and inflammatory cytokine release in sepsis patients (28). Toll-like receptors (TLRS) are an important class of protein molecules involved in non-specific immunity. Chen et al. systematically reviewed the complexity of using Toll-like receptors to intervene in sepsis (29).

Programmed cell death protein 1 (PD-1)/programmed death ligand-1 (PD-L1) immune checkpoint blockade has successfully treated cancer. Because the immune paralysis of sepsis involves depleted T lymphocytes, Nakamori et al. suggested that the role of PD-1/PD-L1 on innate lymphocyte function and the exosome form of PD-L1 deserves further investigation (30). In addition, pathways associated with macrophage apoptosis were identified. Tumor necrosis factor-α (TNF-α) was a key regulator of innate immunity related to sepsis-induced acute lung injury. Yang et al. confirmed that polymorphonuclear neutrophils stimulated by TNF-α up-regulates the expression of NLR family pyrin domain containing 3 (NLRP3) inflammasome through the nuclear factor-κb signaling pathway, which triggers macrophages for pyroptosis (31).

CLEC5A, MALT1, NAIP, NLRC4, SERPINB1, SIRT1, STAT3, and TLR2 were confirmed as sepsis biomarkers. Yang et al. performed bioinformatics analysis and identified SERPINB1 as a potential biomarker of sepsis (the expression of *SERPINB1* was up regulated in patients with sepsis) (32). Zhang et al. used a weighted co-expression network to analyze and establish 15 mRNAs, including NLRC4, as hub genes of the mRNA-lncRNA-pathway network related to sepsis, and the expression of these genes was up-regulated in sepsis patients (33) *Salmonella typhimurium* is a Gram-negative pathogen that causes diseases ranging from gastroenteritis to systemic infections and sepsis. Related experimental analysis by Naseer et al. confirmed the response of caspase-4-mediated inflammasome of NAIP to Salmonella pathogenic island 1-expressing Salmonella (34). Their study elaborated that high expression of NAIP is sufficient to activate the inflammasome in response to infection under physiological conditions. Shayantan et al. used a machine learning approach to identify 20 differentially expressed genes using gene expression profiles of peripheral blood cells obtained within 24 hours of admission to the pediatric ICU (pICU) and extensive clinical data from 228 septic patients from pICU. Among them, eight genes, including CLEC5A, were confirmed to be closely related to sepsis mortality (35). Activation of CLEC5A by the dengue virus and Japanese encephalitis virus leads to the secretion of pro-inflammatory cytokines (36). In addition, many studies have shown that targeting STAT3 has a broad application prospect in treating sepsis (37). STAT3 was further up-regulated during the transition from healthy to sepsis induction. MALT1 deficiency is a congenital immunodeficiency characterized by recurrent bacterial, viral, and fungal infections. Wang et al. demonstrated a significantly high expression of MALT1 in septic samples, and its high expression was closely related to various organ dysfunction (38). In addition, some studies have confirmed that NEAT1 alleviates sepsis-induced myocardial injury by regulating TLR2/NF-κB signaling pathway (39). NEAT1 can significantly down-regulate TLR2 to improve myocardial injury induced by LPS in mice. Deng et al. demonstrated that SIRT1 could alleviate sepsis-induced acute kidney injury through autophagy activation mediated by Beclin1 deacetylation (40) They found that low SIRT1 expression promotes the development of acute kidney injury. In addition, PCR results showed significant differences in the expression of the other 7 genes between the sepsis samples and the normal samples (*P*<0.05).

To explore the relationship between eight diagnostically relevant genes and the immune microenvironment and immune checkpoints, we applied CIBERSORT to estimate the abundance of 22 immune cells. We found that some immune cells were significantly different between the two groups. Xiao et al. elucidated the role of regulatory B cells in the pathogenesis of sepsis (41). Chaturvedi et al. showed that CD8+ T cells have a substantial predictive value for hemophagocytic lymphohistiocytosis versus early sepsis or healthy controls (42). Furthermore, another study suggested that post-sepsis immunosuppression depends on the regulation of mTOR/IFN-γ in NK cells by NKT cells (43). M1 macrophages increase endothelial permeability and enhance p38 phosphorylation in sepsis through PPAR-γ/CXCL13-CXCR5 (44). Mast cells exacerbate sepsis by inhibiting phagocytosis of peritoneal macrophages (45). Neutrophils can interfere with pulmonary microcirculation in sepsis-induced acute lung injury (46).

Our analysis also showed that multiple immune checkpoints were significantly expressed in sepsis. A review by Richendrfer et al. suggested that proteoglycan 4 may enter cells through the CD44 receptor, act on the receptor, and prevent downstream processes of inflammation (47). Wang et al. showed that up-regulation of BTLA expression in bone marrow dendritic cells was associated with treatment outcomes in neonatal sepsis (48).

The present study has some similarities with Wang’s study (49), namely, mining the role of pyroptosis-related genes in sepsis patients and constructing a diagnostic model. In addition, both these studies discussed the immune landscape of hub genes. However, the two papers differed in the following ways. Firstly, we aimed to identify genes associated with sepsis diagnosis using machine learning approaches. To this end, various machine learning methods were used for gene importance assessment and classifier design. However, that paper focused on constructing nomogram models to evaluate the predictive value of clinical factors and genes for sepsis risk. Secondly, the regulatory relationship of the mined genes was queried, and the regulatory network of diagnosis-related genes and miRNAs was constructed. Furthermore, DGIdb was applied to identify potential drugs or molecular compounds that could reverse the expression of diagnosis-related genes. Finally, experimental verification of the mined diagnosis-related genes was carried out.

This study has some limitations. First, the study has a small sample size. Therefore, we started collecting samples from patients with sepsis to investigate these findings further. Secondly, the specificity of the selected pyroptosis-related genes in diagnosing sepsis needs further experimental verification and discussion, especially comparing other inflammatory diseases and sepsis. Finally, given the complexity of the pathological process of sepsis, and to further reduce the application scenario and scope of this experimental results, the following experiments will further verify the different stages of sepsis, e.g., taking suspected sepsis (5), sepsis, and septic shock as separate experimental groups, which can better divide the pathological process of sepsis, which we plan to perform in our next study.

## 5 Conclusion

In this paper, the potential value of diagnosis-related genes in the early diagnosis of sepsis was discussed using machine-learning methods based on the genes related to pyroptosis. In addition, various bioinformatics analysis methods were used to identify the differences between these genes in immune cells and checkpoints. However, different subtypes of sepsis may significantly differ in prognosis and survival. In future research, we plan to further analyze the differences between different subtypes of sepsis patients through various clustering algorithms to provide new insights for precision medicine.

## Data availability statement

The original contributions presented in the study are included in the article/**Supplementary Material**. Further inquiries can be directed to the corresponding author.

## Ethics statement

The studies involving human participants were reviewed and approved by Beijing Tsinghua Changgung Hospital. The patients/participants provided their written informed consent to participate in this study.

## Author contributions

XW performed the major work and drafted the manuscript. ZG, ZiyW, HL, FC and ZiwW participated in revising the manuscript. ZhoW conceptualized, supervised, and revised the manuscript. All authors contributed to the article and approved the submitted version.
