# Supplementary material for: Diagnostic and predictive values of pyroptosis-related genes in sepsis
Source: Front Immunol. 2023 Feb 2;14:1105399. doi: 10.3389/fimmu.2023.1105399 (PMC9932037; doi:10.3389/fimmu.2023.1105399)
Supplement: Supplementary file 1 [file DataSheet_1.pdf]

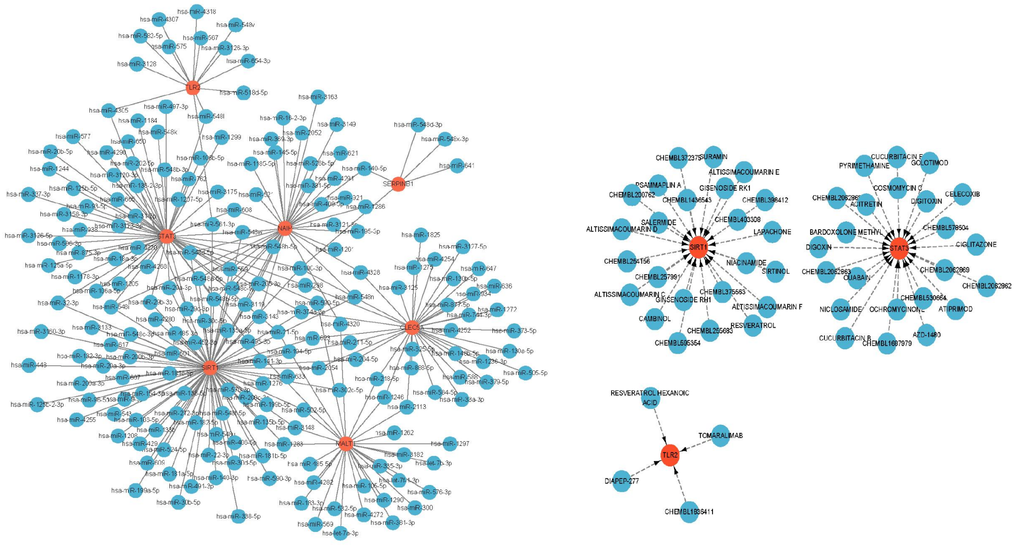

A B

Figure S1 A. Diagnosis-associated gene and miRNA network. B. Gene-drug interaction network.

Table S1. Demographic information for the Sepsis group and the Control group

Parameters	Control	Sepsis	*P*
**Gender**			0.079
Male	4	1	
Female	2	5	
**Ages**			0.003
≥60	0	5	
18-60	6	1	
≤18	0	0	
**Infection Source**			N
Lung	0	4	
Urinary tract	0	1	
Skin or Soft tissues	0	0	
Other	0	1	
**Comorbidities**			0.003
Yes	0	5	
No	6	1	
**ΔSOFA Scores**			0.002
0-2	6	0	
≥2	0	6	
**Lactate levels[a] (mmol/L)**	0.70±0.25	3.97±1.15	0.001

[a] Lactate levels: Mean ± standard deviation, measurement time point: Sepsis group: within 24h after diagnosis of sepsis; Control group: the day of physical examination

Table S2. Primer list

Index	Primer names	Upstream base sequence	Downstream base sequence	Size of product
1	SEPINB1	CGGCCTGTCGGTTTTCAC	TCTCACTCAACGCCAGGAAC	83
2	NLRC4	GCTTCATGGGAAAAGGCTGC	GCTGAAATCCCGGAGTGTGA	147
3	NAIP	TTCTGAACGGCCAGAAGACC	AGGGGAGGGGAGTTGACTTT	115
4	CLEC5A	AGCAGAGCCACCCTAGATCA	GGGAGAAGGATGAAGGCCAG	140
5	STAT3	CTGTGCGTATGGGAACACCT	GCTGAGGCAAGGTGGTTTTG	95
6	MALT1	GCACCAGGGTAGCAGATAGAA	ATCCTGGCTCTGCCCTTACTA	168
7	TLR2	TGGCCACAAAAGGCATTCTC	TGGGGAGTGCCCCAAATACT	95
8	SIRT1	TCACTGTGGTAGAGCTTGCAT	GGCCTGTTGCTCTCCTCATT	158
9	GAPDH	GGAGCGAGATCCCTCCAAAAT	GGCTGTTGTCATACTTCTCATGG	197
